# Susceptibility Evaluation to Fire Blight and Genome-Wide Associations within a Collection of Asturian Apple Accessions

**DOI:** 10.3390/plants12234068

**Published:** 2023-12-04

**Authors:** Belén García-Fernández, Ramon Dolcet-Sanjuan, Diego Micheletti, María José Antón-Díaz, Cristina Solsona, Mercedes Fernández, Xavier Abad, Enrique Dapena

**Affiliations:** 1 Regional Service for Agrofood Research and Development (SERIDA), Ctra AS-267, PK 19, 33300 Villaviciosa, Spain; mjanton@serida.org (M.J.A.-D.); mercedfr@serida.org (M.F.); 2 IRTA Fruitcentre, PCiTAL, Parc AgroBiotech-Gardeny, 25003 Lleida, Spain; ramon.dolcet@irta.cat (R.D.-S.); cristina.solsona@irta.cat (C.S.); 3 Research and Innovation Center, Edmund Mach Foundation, 38098 San Michele all’Adige, Italy; diego.micheletti@fmach.it; 4 IRTA-CReSA, Centre de Recerca en Sanitat Animal, 08193 Bellaterra, Spain; xavier.abad@irta.cat

**Keywords:** *Malus domestica*, *Erwinia amylovora*, cider apple, apple bank germplasm, phenotypic evaluation, local cultivars, artificial inoculations, genome-wide association studies, apple breeding, putative candidate genes

## Abstract

Fire blight, caused by *Erwinia amylovora*, is one of the most devastating apple diseases. The selection of cultivars of low susceptibility and the study of the genetic mechanisms of the disease play important roles in fire blight management. The susceptibility level to fire blight was evaluated in 102 accessions originating from Asturias, a cider-producing region located in the north of Spain with a wide apple germplasm. Evaluations took place under quarantine conditions using artificial inoculations of grafted plants. The results revealed wide variation in susceptibility responses and low-susceptible cultivars were identified. In addition, 91 cultivars were genotyped using the Affymetrix Axiom^®^ Apple 480 K SNP array to conduct genome-wide association studies (GWAS). A statistically significant signal was detected on chromosome 10 using the multi-locus mixed model (MLMM). Two genes were identified as major putative candidate genes: a TIR-NBS-LRR class disease protein and a protein containing a development and cell death (DCD) domain. The outcomes of this study provide a promising source of information, particularly in the context of cider apples, and set a starting point for future genetic and breeding approaches.

## 1. Introduction

The domesticated apple (*Malus domestica* Borkh.) is one of the most widely cultivated tree fruits in temperate regions. Apple trees can be easily propagated and they bear quality fruit, which is considered one of the top functional foods [1,2]. Accordingly, global production for the year 2021 was estimated at 93 million metric tons [3]. Of the more than 500,000 tons produced in Spain that year, approximately 15% were cider apples and 85% were dessert ones [4].

The Gram-negative bacterium *Erwinia amylovora* [5,6] is the causative agent of fire blight, which is one of the most damaging apple diseases. Since its detection in the United States more than 200 years ago [7], it has been detected in many parts of the world. The consequences of fire blight infections can be devastating to apple orchards, as a severe outbreak can disrupt their production for years [8]. In 2007, European apple production suffered particularly severe economic losses due to elevated inoculum pressure and favorable warm temperatures during the flowering period [9].

The control of fire blight is a challenge due to the number of tissues that are susceptible to infection by the bacteria and the reduced number of management tools to control it [10]. Moreover, many of the varieties that were introduced in the last few decades (e.g., ‘Fuji’, ‘Gala’, ‘Ginger Gold’ or ‘Pink Lady’) are more susceptible to the bacteria than most older cultivars [10]. The application of antibiotics, mainly streptomycin, was one the most effective chemical control against *E. amylovora* [10,11]. However, the identification of streptomycin-resistant strains has reduced its effectiveness [10,11,12,13,14,15,16,17]. On the other hand, due to the risks of using antibiotics, their application was banned in many countries for sustainability and consumer-friendly issues [18]. In this sense, breeding for host resistance is an essential component of the integrated fire blight management strategy [19].

Several screenings have been performed in *Malus* spp. to determine host susceptibility of different accessions [20,21,22,23,24,25]. These evaluations are usually carried out by field assessments or through artificial inoculations under controlled conditions in areas where the bacteria is widely spread [26]. Nevertheless, they must be conducted under quarantine conditions and, consequently, through artificial inoculations in areas categorized as protected zones due to the quarantine status of the disease [26,27]. In this situation, the most commonly used inoculation methods are based on artificial terminal shoot or leaf infection [25].

There are also numerous works focused on unraveling the genetic basis of fire blight resistance. QTL mapping approaches using wild species revealed loci associated with a strong resistance located on linkage group 3 (LG3; LG number also corresponds to chromosome number) of *Malus* × *robusta* 5, on LG10 of *Malus fusca* and on LG12 of *Malus* ‘Evereste’, *Malus floribunda* 821 and *Malus* × *arnoldiana* [28,29,30,31]. Regarding domesticated cultivars, the most important one corresponds to a major QTL located on LG7 of the cultivar ‘Fiesta’, which was reported by two independent studies developed by Calenge et al. [32] and Khan et al. [33]. A complementary study carried out by Khan et al. validated this QTL and checked its application in marker-assisted selection [34]. Furthermore, other QTLs have also been reported in different QTL mapping studies [32,35,36,37,38]. Additionally, a recent genome-wide association study (GWAS) developed by Thapa et al. [39] also reported significant marker-trait associations by rating both shoot and blossom fire blight infection severity.

In addition to being the Spanish region with the highest cider production and a large cider-making tradition [40,41], Asturias, located in the north of the country, also has a rich apple diversity, especially of cider cultivars [42,43]. The majority of this diversity is represented by the more than 500 local accessions that are preserved in the Apple Germplasm Bank of Asturias, maintained by the SERIDA (Regional Service for Agrofood Research and Development, Villaviciosa, Spain). The characterization of this plant material has enabled the selection of cultivars of interest for the cider sector [44,45,46,47,48,49,50]. Indeed, out of the 76 varieties included in the Protected Designation of Origin (PDO) “Sidra de Asturias”, 58 are Asturian cultivars and 18 are selections from the cider-apple breeding program developed by the Fruit Research Unit of the SERIDA [51,52,53,54]. Favored by wet and mild springs, Asturias remains free of fire blight and maintains the status of a protected zone [55]. Despite this, it is important to make efforts in the evaluation, selection and breeding of local apple cultivars as prevention and management strategies against this disease.

Taking this context into account, the goals of this work were to evaluate the susceptibility level against *E. amylovora* among local accessions and to conduct GWAS in order to determine phenotype–genotype associations.

## 2. Results

### 2.1. Phenotypic Evaluation

#### 2.1.1. Pathogenicity Test

ANOVA did not reveal differences in virulence among isolates (F_2,69_ = 1.02; *p*-value = 0.31). Nonetheless, the isolate FB74 was selected for the phenotypic evaluation since it showed the highest mean area under the disease progress curve (AUDPC) value when compared to FB45 and EW07 (Table 1).

#### 2.1.2. Evaluation of Plant Material Susceptibility

**Figure 1:**
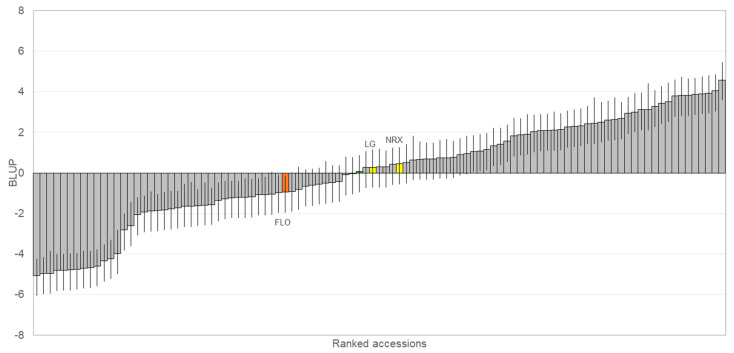
Distribution of best linear unbiased prediction (BLUP) values ± standard deviation. Accessions were ranked in increasing order, from the lowest to the highest susceptibility levels. The orange column represents ‘Florina’ (FLO) and the yellow columns correspond to ‘Lagar’ (LG) and ‘No Raxao’ (NRX) (identical genotypes).

Figure 1 shows the distribution of the accessions according to their best linear unbiased prediction (BLUP) ± standard deviation. They were ordered from the lowest to the highest BLUP value; in other words, from the lowest to highest susceptibility level.

‘Florina’ (FLO), international control (see Section 5.1.2.), was represented in orange. ‘No Raxao’ (NRX) and ‘Lagar’ (LG) (identical genotypes) [56] had BLUP values of 0.43 and 0.28, respectively (represented in yellow in Figure 1).

Phenotypic evaluation revealed a wide variation in host responses. Focusing on those cultivars included in the PDO, low susceptibility (BLUP < −4) were detected in varieties such as ‘Carrandona’, ‘Arbeya’, ‘Durona de Tresali’, ‘Xuanina’ and ‘Lin’. At the other end of the distribution (BLUP > 3), highly susceptible cultivars like ‘Durón Encarnado’, ‘San Roqueña’, ‘Limón Montés’, ‘Perico’, ‘Fuentes’, ‘Regona’ or ‘Meana’ were found. On the other hand, ‘Panquerina’, ‘Durón de Arroes’, ‘Solarina’, ‘Repinaldo de Hueso’ and ‘Ernestina’ are examples of cultivars ranked as moderately susceptible (BLUP from −0.5 to 0.5). BLUP values, as well as the number of replicates that were evaluated per accession, are compiled in Table S1.

### 2.2. Genotyping and Population Genetic Structure

All the diploid genotypes hybridized (90 local + ‘Florina’; Table S1) passed the quality controls of the Axiom™ Analysis Suite. In addition, 243,495 SNPs were classified as batch alle consistency (BAC)-passed, PolyHighResolution and very robust. Of these, 28,954 SNPs were discarded because of a minor allele frequency (MAF) lower than 5%. Genotyping resulted in a final set of 91 genotypes and 214,541 SNPs to implement phenotype–genotype associations.

**Figure 2:**
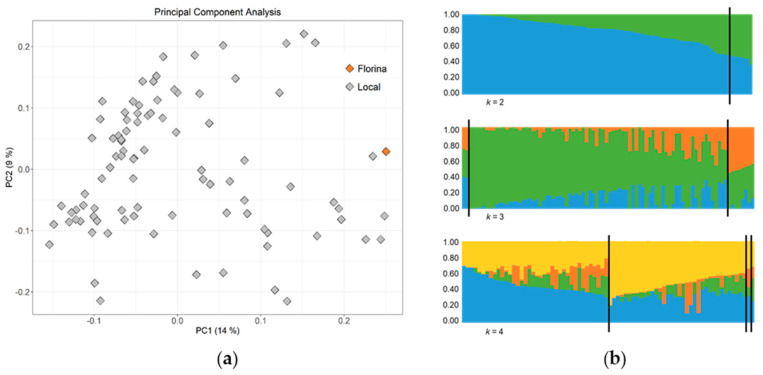
Population genetic structure analyses. (**a**) Principal Component Analysis (PCA). Principal Component 1 (PC1) was plotted against Principal Component 2 (PC2). The percentage of variance explained by each one is shown in parentheses; (**b**) Distribution of the genotypes after dividing the collection into 2, 3 and 4 subpopulations (*k*).

Population structure analyses are plotted in Figure 2. Principal component analysis (PCA) did not reveal separate clusters (Figure 2a). The amount of variance explained by the first two components (PC1 and PC2) was 14% and 9%, respectively. Bayesian clustering (Figure 2b) also reflects high admixture within the membership coefficients (*Q*), even when the number of subpopulations was two (*k* = 2). Similarly, the STRUCTURE HARVESTER revealed the highest Delta *K* value for *k* = 2. An in-depth study of the population genetic structure discarded strong clustering in the collection.

### 2.3. Phenotype–Genotype Associations

**Figure 3:**
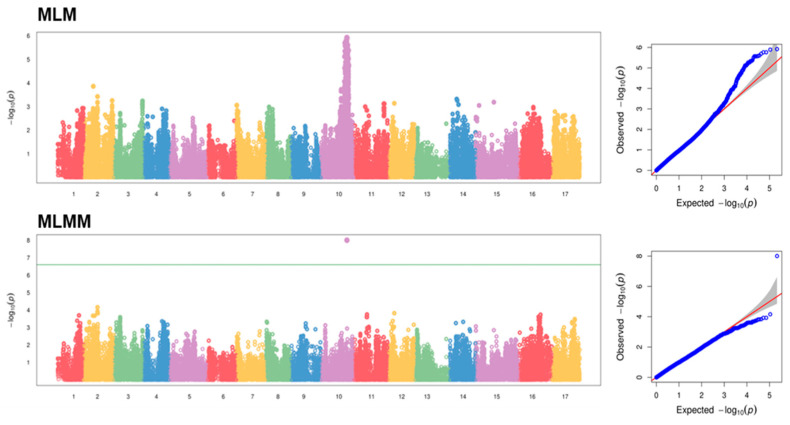
Manhattan plots and corresponding Q-Q plots for the mixed linear model (MLM) and the multiple loci mixed model (MLMM) after conducting genome-wide association analysis (GWAS) for fire blight. Different chromosomes are represented by different colors. The horizontal green line in the Manhattan plots indicates the threshold at a significant level of 5% after Bonferroni multiple test correction. Q-Q plots compare the expected value for the −log_10_(*p*-value) vs. the observed one for each SNP (blue point). The red line indicates a hypothetical distribution without association.

A significant signal was detected using the multi-locus mixed model (MLMM) at the bottom of chromosome 10 (Figure 3). The significant marker was AX-115639581 (*p*-value = 9.92 × 10^−9^; −log_10_(*p*-value = 8.00), located on chromosome 10 position 32,827,282 of the ‘Golden Delicious’ doubled-haploid genome (GDDH13) v. 1.1. Even though no markers exceeded the significance threshold of 6.6 using the mixed linear model (MLM), a convincing peak was identified in the same window region as MLMM. Indeed, the −log_10_(*p*-value) of AX-115639581 in MLM was 5.9. For both MLM and MLMM, the Q-Q plot indicated a good fit of the model, as shown in Figure 3.

**Figure 4:**
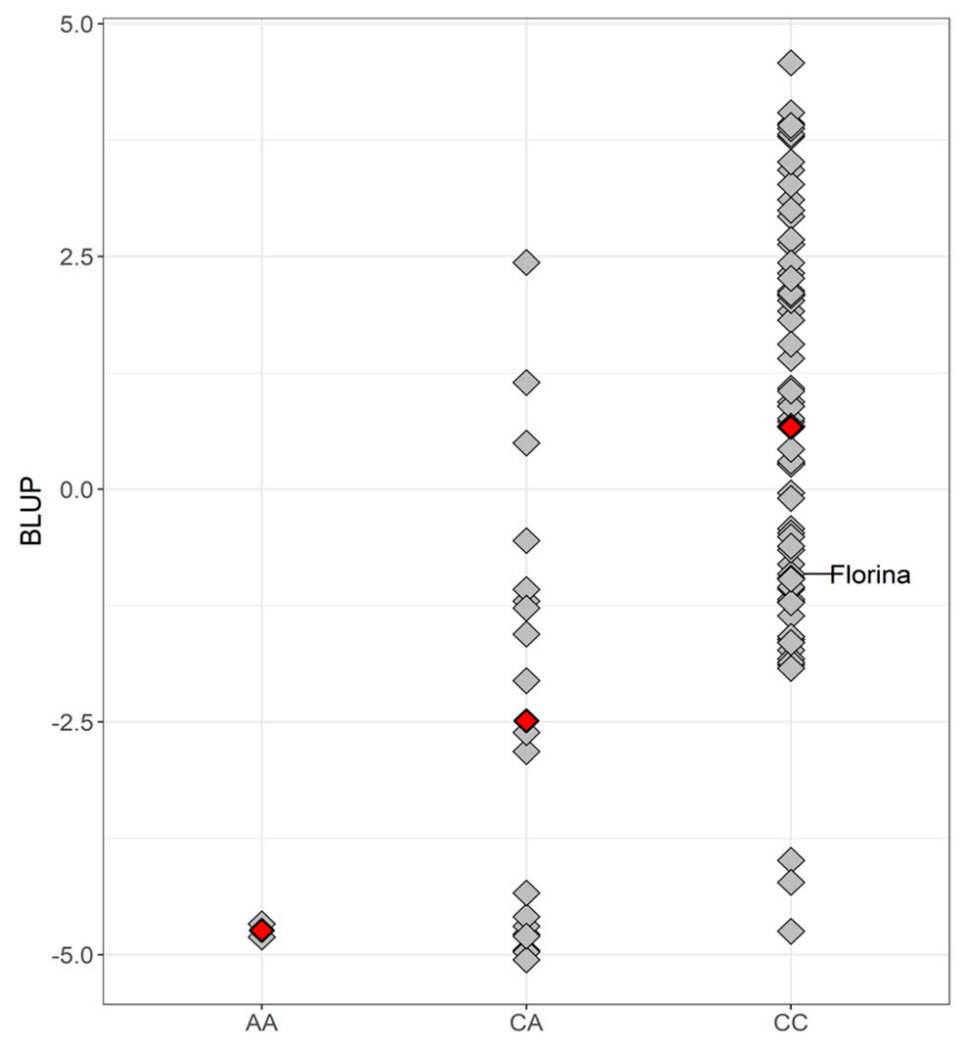
Scatter plot of the best linear unbiased prediction (BLUP) results grouped by allele (C/A) of AX-115639581. The grey rhombuses represent each cultivar while the red ones represent the mean of each group.

Considering the MLMM, the effect and the percentage of phenotypic variance explained (*R*^2^) by AX-115639581 were 3.22 and 55.61%, respectively. Figure 4 represents the scatter plot of the BLUP values grouped by allele (C/A) of AX-115639581. Seventy genotypes were homozygous CC, 19 were heterozygous CA and 2 were homozygous AA (MAF = 0.13) (Table S1). The ANOVA test revealed significant differences among means (red rhombus; *p*-value < 0.001), although a wide phenotypic variability can be observed within the heterozygous and homozygous CC groups (Figure 4).

Of the five cultivars included in the PDO that showed low BLUP values (see Section 2.1.2), ‘Carrandona’ was homozygous AA, ‘Arbeya’ and ‘Xuanina’ were heterozygous CA, and ‘Durona de Tresali’ and ‘Lin’ were the other homozygous CC.

## 3. Discussion

In accordance with the global context of fostering sustainability, the evaluation, selection and use of low-susceptible apple cultivars represents an important advance in fire blight management.

In this sense, evaluation using grafted plants was chosen despite requiring more space since it has been reported to show more reproducibility, less variability and high resolution compared to detached leaves [22,57,58]. Moreover, Ruz et al. [26] found, while working in grafted pear plants, that the most effective inoculation method was cutting with scissors compared to pricking with clamps, local infiltration and painting onto the leaves.

Despite having used the same inoculum concentration, the same inoculation procedure, and trying to adjust the growth rates among replicates, the standard deviation within the same genotypes was moderate, which highlights the importance of keeping homogeneous conditions while evaluating fire blight infection. In this case, the observed results for ‘Florina’ fell between those reported by Le Roux et al. [35] (moderate susceptibility) and those described by Le Lezec et al. [59] (low susceptibility). Furthermore, the strong similarity between the two genetically identical accessions, ‘No Raxao’ and ‘Lagar’, provides robustness to the assessment.

The phenotypic evaluation carried out in this study allowed the assessment of the susceptibility to fire blight in 102 Asturian-local accessions, leading to the identification of five low-susceptible accessions that included cultivars protected by PDO “Sidra de Asturias”. Indeed, two of them, ‘Durona de Tresali’ and ‘Xuanina’, constitute 20.5% of the total production of PDO [60].

Nonetheless, it is important to note that different strains of *E. amylovora* have been found to exhibit varying degrees of virulence and cultivars have shown differential susceptibility to these bacterial strains, as has been reported for *Malus* × *robusta* 5 [10,22,37,61,62,63,64,65]. The majority of the cultivars included in PDO that were previously analyzed (e.g., ‘Xuanina’, ‘Durona de Tresali’, ‘Regona’, ‘Meana’ or ‘San Roqueña’) [66] showed consistent results between both evaluations. However, ‘Perico’ exhibited a different response level, which could be caused by this strain-dependent susceptibility. Specifically, ‘Perico’ was of low susceptibility, whereas in this study it showed a high infection level. In light of these findings, it is wise, as suggested by Martínez-Bilbao et al. [22], to continue the evaluation of cultivars by testing them against different strains. This recommendation, along with the use of multiple replicates due to variability, is also endorsed in the present study.

Chromosome 10 has been associated with fire blight resistance in previous studies. In particular, Thapa et al. [39] identified QTLs in the middle region of LG10, but they are distinct from the region identified here. Emeriewen et al. [67] aligned the predicted sequence of the *FB_Mfu10* gene [18,30,68] against the GDDH13 genome, revealing the best match in the region between 30,708,564 and 30,712,095, also on LG10. The marker AX-115639581 is located approximately 2 Mbp downstream from this region, which points to a different source of resistance. After exploring the window region of the marker, two putative-candidate genes were found: *MD10G1232100*, annotated as development and cell death domain protein (DCD); and *MD10G1232400*, annotated as TIR-NBS-LRR class disease protein. Both are located downstream of the marker, 12 Kbp and 29 Kbp, respectively [69]. TIR-NBS-LRR consists of a leucine-rich repeat domain (LRR), a nucleotide binding site (NBS) and an amino-terminal domain with homology to the Toll and interleukin 1 receptors (TIR) and is one of the major classes of plant resistance genes (*R*-genes) [70]. Overexpression of genes encoding TIR-NBS-LRR was detected in transcriptome analyses during apple infections with *E. amylovora* [39,71,72]. On the other hand, the DCD domain, which is composed of approximately 130 amino acid residues, can be found throughout the plant kingdom [73]. It seems that DCD could mediate signaling in plant programmed cell death by pathogens [73].

Since the MLM emerged [74], MLM-based GWAS have been widely conducted. However, multi-locus models are better models when working with polygenic traits [75,76], the case of this study. Furthermore, the lack of statistical significance observed in the MLM could be partially explained by the reduced sample size or by the correction for multiple testing, which is often too conservative [76,77,78]. On the other hand, unexpected results between phenotype and genotype (Figure 4) can be due to recombination events in the region or strain-specific resistance.

## 4. Conclusions

Deciphering the genomic basis of quantitative variation is crucial to understanding evolution and accelerating plant breeding [79]. Although the potential impact of strain-specific resistance and the increase in sample size should be considered for further analyses, this study sets a starting point for future genetic approaches.

The identification of a genomic region associated with fire blight can be used to perform a marker-selection approach, which, in turn, can decrease both the time and money required for developing and selecting desirable genotypes [80]. Moreover, association mapping using populations of unrelated individuals can be combined with QTL analysis in bi-parental and multi-parental crosses to reduce the confidence interval of the associated regions, and complementing studies such as gene editing could validate the role of the putative candidate genes during fire blight infections [81,82,83].

In conclusion, the findings of this study not only offer promising information but also serve as a valuable resource for the selection of optimal cultivars for apple production and for their integration into breeding programs, especially within cider apple growing.

## 5. Materials and Methods

### 5.1. Phenotypic Evaluation

#### 5.1.1. Plant Material and Culture Conditions

Susceptibility was evaluated on potted plants in 103 accessions preserved in the Apple Germplasm Bank of Asturias (Table S1). Of them, 42 local cultivars are included in the PDO “Sidra de Asturias”, 60 accessions are representative of the local diversity and the last one corresponds to the international cultivar ‘Florina’.

Phenotypic evaluation was performed by inoculation of growing shoots on potted plants over two years: 2019 and 2021. For each year, eight 1-year-old shoots per accession were whip-grafted onto ‘M7’ EMLA apple rootstock and potted on 770 mL containers with substrate Exclusive (Gebr.Brill Substrate GmbH & Co. KG, Georgsdorf, Germany). Dormant plants were placed in a cold greenhouse until they reached a length of 5 cm. Then, they were transferred to a cold chamber set at 14 °C and 16/8 h light/dark photoperiod (200 μmol·m^−2^·s^−1^), when they continued to grow until they were moved to the high-biocontainment box (BSL3) of the IRTA—CReSA (Campus of the Autonomous University of Barcelona, Cerdanyola del Vallès, Cataluña, Spain).

Plants that failed to achieve a growth of 10 cm were not moved to the high-biocontainment box and they were not evaluated. Moreover, the maximum number of replicates per accession and year was set at six and the minimum number at three, considering those accessions with fewer than three valid replicates unsuccessful for that year. Plant material was inoculated one week after moving to the high-biocontainment box, where culture conditions were set at 23 °C, high relative humidity (≈100%) and 16/8 h photoperiod with 300 mmol·m^−2^·s^−1^ of photosynthetically active radiation (PAR) (Figure S1). Plants were fertilized with Proturf^®^ Active 15-5-15 (ICL Ibéria, Súria, Spain) and watered every two days with tap water at soil level, avoiding shoot or leaf watering.

All plant material was evaluated in a single batch in 2019, and it was divided into two batches due to different growth rates in 2021. Plant material was randomly distributed by blocks in the three batches.

#### 5.1.2. Pathogenicity Test

Pathogenicity was performed on three different isolates identified as *E. amylovora* by nucleic acid analysis of the 1100 pb rDNA 16S fragment at the Laboratory of Instrumental Techniques of the University of León. FB74 and FB45 were isolated from an infected apple orchard in Catalonia (Spain) and stored at the IRTA—CreSA. EW07 was provided by the Department of Engineering and Agricultural Science (University of León, León, Spain).

Eight apple cultivars, ‘Florina’ and seven local ones, were used to test the pathogenicity of the isolates. The local cultivars ‘Collaos’, ‘De la Riega’, ‘Limón Montés’, ‘Meana’, ‘Regona’, ‘Solarina’ and ‘Xuanina’ were selected because they previously showed different levels of susceptibility against the bacteria [66] and ‘Florina’ was used as a control for low-moderate susceptibility [35,59]. All of them, along with two genetically identical individuals (‘No Raxao’ and ‘Lagar’) [56], were also used as reference in the phenotypic evaluation of the collection analyzed.

The level of pathogenicity was assessed following the same protocol described above for culture conditions and the same workflow described below for inoculum preparation, inoculation and evaluation.

#### 5.1.3. Inoculum Preparation and Artificial Inoculation

Isolate FB74 stored at −80 °C was sown using the three-phase streaking pattern (T-streak) in fresh LB media and incubated at 25 °C for 48 h. A suspension of 108 cfu/mL was prepared by resuspending the isolated colonies in sterile water. Adjustment was performed using a viable absorbance relationship previously prepared (absorbance = 0.1 at λ = 620 nm) [84]. The inoculum was prepared two hours before use and was stored at 4 °C until then.

Inoculation was performed by cutting the first completely unfolded leaf using scissors previously immersed in the bacterial suspension [61]. Two cuts per leaf were performed at different positions and on each side of the mid-rip, taking care not to break it.

#### 5.1.4. Evaluation of Plant Material Susceptibility

**Figure 5:**
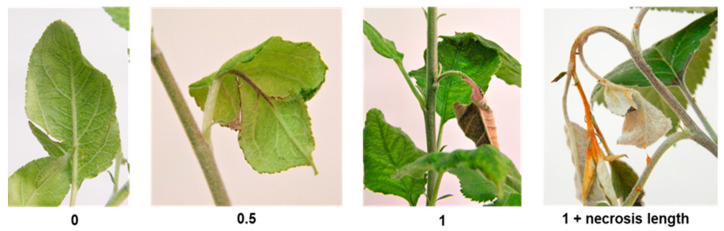
Representative examples of the scale used to measure necrosis progression: 0—no visible symptoms; 0.5—necrosis only affected veins of the inoculated leaf; 1—necrosis reached the petiole of the inoculated leaf; 1 + necrosis length—necrosis reached the stem of the plant.

Susceptibility was recorded 7, 14 and 21 days post-inoculation by dividing necrosis progression by shoot length. Necrosis progression was measured using the scale described by Calenge et al. [32] (Figure 5): (i) 0—no visible symptoms; (ii) 0.5—necrosis only affected veins of the inoculated leaf; (iii) 1—necrosis reached the petiole of the inoculated leaf; (iv) 1 + necrosis length—necrosis reached the stem.

### 5.2. Genotyping

To generate the genotypic data, the genomic DNA of 91 diploid accessions (90 local + ‘Florina’; Table S1) was hybridized on the Affymetrix Axiom^®^ Apple 480 K SNP array [85] following the manufacturer’s protocol at the genotyping platform of FEM (Fondazione Edmund Mach, San Michele all’Adige, Italy). Raw data were processed with the “Best Practices” workflow for the diploid clustering method in the Axiom™ Analysis Suite v. 5.2. A BAC analysis was performed to correct for eventual batch effects. Of the set of SNPs that were classified as PolyHighResolution by the Axiom™ Analysis Suite and passed the BAC analysis, those defined as very robust markers by Bianco et al. [85] were extracted and filtered by a MAF threshold of 0.05 using PLINK v. 1.9 [86]. The physical position of the SNPs was determined by blasting the probes against the reference genome GDDH13 v. 1.1 [87] and assigning the ambiguous-positioned markers to a fictive chromosome 0.

#### Population Genetic Structure

The population structure was studied using both PCA and Bayesian clustering. PCA was calculated using the PLINK software, which was also used to prune the set of SNPs using the “indep” option. Pruning was performed by setting a window size of 1 Mbp, a step size of 10 and linkage disequilibrium (LD) measured as *r*^2^ below 0.5. The subset of LD-pruned markers was used in the software STRUCTURE v. 2.2 [88], setting a burn-in length of 10,000, a run length of 50,000 and ranging the number of subpopulations (*k*) from 1 to 6. After running seven replicates for each *k*, STRUCTURE HARVESTER v. A.2 [89] was used to estimate the most probable number of *k*.

### 5.3. Statistical Analyses and Phenotype–Genotype Associations

Susceptibility data recorded at 7, 14 and 21 days post inoculation were used to calculate AUDPC values in the ‘agricolae’ package v. 1.3-6 [90]. Pathogenicity differences were analyzed by performing a one-way ANOVA analysis at a significance level of 5% with the AUDPC values.

Phenotypic results were adjusted by fitting a mixed linear model using the ‘*lme4*’ package v. 1.1-34 [91]. “Accession” and “batch” were considered random and fixed effects, respectively. BLUP values of the random effects were also extracted using the ‘*lme4*’ package.

Phenotype–genotype associations were conducted using the MLM and MLMM methods implemented in the ‘*GAPIT*’ package v. 3.1.0 [92], setting BLUP values as phenotypic-input data. Both the kinship matrix and the first three principal components (PCs) were included in the association model. The kinship matrix was calculated in TASSEL v. 5.2.86 [93] using the “Centered_IBS” method [94]. After applying the Bonferroni multiple test correction, the -log_10_ threshold for significant associations was set to 6.6 (*p*-value = 2.33 × 10^−7^) at a significant level of 5%. Genes within windows of 100 Kbp in both directions of a significant marker were explored using the JBrowser tool hosted on the Genome Database for *Rosaceae* (GDR) website [69].

All analyses were implemented in the R software environment v. 4.3.1 [95] and the ‘*ggplot2*’ package v. 3.4.2 [96] was used to visualize the PCA analysis and BLUP results grouped by allele of AX-115639581.

## Figures and Tables

**Table 1:** Isolate name, origin and area under disease progress curve (AUDPC) values of the *Erwinia amylovora* isolates. The same letter next to AUDPC values (a) means non-significant differences based on the ANOVA analysis (*p*-value < 0.05).

Isolate	Origin	AUDPC
EW07	Department of Engineering and Agricultural Science (University of León, León, Spain)	5.77 a
FB45	IRTA—CReSA (Campus of the Autonomous University of Barcelona, Cerdanyola del Vallès, Cataluña, Spain)	6.89 a
FB74	IRTA—CReSA (Campus of the Autonomous University of Barcelona, Cerdanyola del Vallès, Cataluña, Spain)	7.54 a

## Data Availability

Data sharing is available upon request.

## Supplementary Materials

The following supporting information can be downloaded at: https://www.mdpi.com/article/10.3390/plants12234068/s1, Table S1: Compilation of the plant material information and phenotypic results. The name, protection status from the Protected Designation of Origin (PDO) “Sidra de Asturias” and if they were used to implement phenotype–genotype associations is reported for each accession. The number of replicates evaluated, best linear unbiased prediction (BLUP) value and genotypic information for marker AX-115639581 is also reported for each one; Figure S1: Image of potted plants 21 days after inoculation in the culture conditions of the high-biocontainment box (BSL3) at the IRTA—CReSA.
