# Supplementary material for: Susceptibility Evaluation to Fire Blight and Genome-Wide Associations within a Collection of Asturian Apple Accessions
Source: Plants (Basel). 2023 Dec 4;12(23):4068. doi: 10.3390/plants12234068 (PMC10708232; doi:10.3390/plants12234068)
Supplement: Supplementary file 1 [file plants-12-04068-s001.zip › plants-2738037-supplementary.pdf]

# Supplementary material for "Susceptibility Evaluation to Fire Blight and Genome-Wide Associations within a Collection of Asturian Apple Accessions"

**Belén García-Fernández [1,*], Ramon Dolcet-Sanjuan [2], Diego Micheletti [3], María José Antón-Díaz [1], Cristina Solsona [2], Mercedes Fernández [1], Xavier Abad [4] and Enrique Dapena [1,*]**

[1] Regional Service for Agrofood Research and Development (SERIDA), Ctra AS-267, PK 19, 3300 Villaviciosa, Spain; mjanton@serida.org (M.J.A.-D.); mercedfr@serida.org (M.F.)
[2] IRTA Fruitcentre, PCiTAL, Parc AgroBiotech-Gardeny, 25003 Lleida, Spain; ramon.dolcet@irta.cat (R.D.-S.); cristina.solsona@irta.cat (C.S.)
[3] Research and Innovation Center, Edmund Mach Foundation, 38098 San Michele all'Adige, Italy; diego.micheletti@fmach.it
[4] IRTA-CReSA, Centre de Recerca en Sanitat Animal, 08193 Bellaterra, Spain; xavier.abad@irta.cat
* Correspondence: bgarcia@serida.org (B.G.-F.); edapena@serida.org (E.D.)

**Table S1**. Compilation of plant material information and phenotypic results. The name, protection status from the Protected Designation of Origin (PDO) "Sidra de Asturias", and if they were used to implement phenotype–genotype associations is reported for each accession. The number of replicates evaluated, Best Linear Unbiased Prediction (BLUP) value and genotypic information for marker AX-115639581 is also reported for each one.

| **Name¶*** | **Replicates** | **BLUP** | **Genotype** | **Name¶*** | **Replicates** | **BLUP** | **Genotype** |
|---|---|---|---|---|---|---|---|
| Amandi* | 12 | −1.21 | CC | **Mariñana*** | 6 | −1.65 | CC |
| **Carrió** | 11 | −1.78 | - | Parda Carreño | 11 | 2.28 | - |
| **Cristalina*** | 8 | 1.91 | CC | Pardina* | 11 | −1.36 | CC |
| Loroñesa | 5 | 0.65 | - | Ricu* | 5 | −2.61 | CA |
| Mingán* | 12 | 3.79 | CC | Tomate* | 10 | −0.10 | CC |
| Panera* | 10 | 1.82 | CC | **Meana*** | 11 | 3.11 | CC |
| **Paraguas** | 10 | 1.34 | - | Obdulina* | 12 | 0.27 | CC |
| **Reineta Caravia*** | 9 | 0.50 | CA | Pachín Torteru* | 10 | 0.67 | CC |
| Reineta Panera* | 9 | 3.81 | CC | Parda Blanquera* | 6 | −1.58 | CC |

| Name[¶*] | Replicates | BLUP | Genotype | Name[¶*] | Replicates | BLUP | Genotype |
|---|---|---|---|---|---|---|---|
| Solafuente* | 9 | −1.20 | CA | Peñaflor | 9 | 0.74 | - |
| Villaviciosa* | 11 | 0.73 | CC | Pepa* | 10 | −1.18 | CC |
| **Arbeya*** | 12 | −4.78 | CA | **Perezosa*** | 10 | −1.07 | CC |
| Brava* | 11 | −1.73 | CC | Rendueles* | 12 | 2.93 | CC |
| Busín* | 5 | −3.99 | CC | Revoltosa* | 12 | −1.08 | CA |
| Calabaza* | 6 | −4.95 | CA | Rozona* | 6 | −1.05 | CC |
| **Collaos*** | 10 | −1.82 | CC | Valsaína* | 12 | −4.34 | CA |
| Chapa* | 12 | 1.40 | CC | Valliniello* | 11 | −2.06 | CA |
| Dulce Alba* | 11 | 0.94 | CC | **Blanquina*** | 12 | −1.22 | CC |
| **Durón de Arroes*** | 12 | −0.42 | CC | **Coloradona*** | 9 | 3.00 | CC |
| Emiliano* | 6 | −0.80 | CC | Reineta de Santirso* | 11 | 2.32 | CC |
| **Fuentes*** | 11 | 3.43 | CC | **Clarina*** | 4 | 2.44 | CA |
| Manuel Isla | 4 | 3.11 | - | **Raxao*** | 12 | −1.92 | CC |
| Mariano* | 6 | −1.64 | CC | Esperiega | 8 | 2.51 | - |
| **Miyeres*** | 12 | −0.55 | CA | Chata Encarnada Mut. | 12 | 0.68 | - |
| Montés de Prau* | 12 | 0.76 | CC | **De la Riega*** | 11 | 2.03 | CC |

| Name¶* | Replicates | BLUP | Genotype | Name¶* | Replicates | BLUP | Genotype |
|---|---|---|---|---|---|---|---|
| **Montoto** | 12 | 1.87 | - | **Verdialona***| 12 | 2.68 | CC |
| **Panquerina*** | 12 | −0.47 | CC | Florina* | 12 | −0.95 | CC |
| **Perico*** | 11 | 3.81 | CC | **Durona de Tresali*** | 12 | −4.75 | CC |
| Pomarón de Benavides* | 12 | 1.15 | CA | **Ernestina*** | 12 | 0.30 | CC |
| **Prieta*** | 12 | 2.09 | CC | **Limón Montés*** | 12 | 3.87 | CC |
| Prieta Antigua* | 6 | 2.63 | CC | Picona Rayada* | 11 | 3.91 | CC |
| No Raxao | 12 | 0.44 | - | **Regona*** | 12 | 3.27 | CC |
| Reineta de Pravia* | 10 | 3.93 | CC | **Solarina** | **12** | 0.06 | - |
| Reineta Parraguesa* | 12 | −2.82 | CA | VPC005* | 12 | −4.67 | AA |
| Reineta Verde* | 12 | 1.08 | CC | VPC007* | 12 | −5.05 | CA |
| **Repinaldo Caravia** | 8 | 2.60 | - | VPC008* | 12 | −4.80 | CA |
| **Repinaldo de Hueso*** | 10 | 0.30 | CC | VPC060* | 12 | 0.89 | CC |
| Repinaldo Picón B.* | 11 | −4.69 | CA | **Beldredo*** | 12 | 1.05 | CC |
| **San Roqueña*** | 12 | 4.04 | CC | **Picón*** | 12 | 2.08 | CC |
| Sombrerín* | 10 | −0.91 | CC | VPC138* | 9 | 2.11 | CC |
| **Sucu*** | 9 | −1.89 | CC | VPC161* | 12 | 1.56 | CC |
| **Teórica*** | 12 | 2.13 | CC | VPC168* | 10 | −1.28 | CA |

| Name¶* | Replicates | BLUP | Genotype | Name¶* | Replicates | BLUP | Genotype |
|---|---|---|---|---|---|---|---|
| Vega* | 12 | −1.56 | CA | **Montés de la Llamera*** | 12 | −0.61 | CC |
| **Verdosa*** | 6 | −0.51 | CC | **Carrandona*** | 12 | −4.81 | AA |
| **Xuanina*** | 12 | −4.59 | CA | VPC322* | 10 | 2.43 | CC |
| Bedriñana* | 12 | −1.86 | CC | Lin* | 9 | −4.22 | CC |
| De Obispo* | 12 | −1.61 | CC | VPC353* | 10 | −0.97 | CC |
| Rayada* | 12 | −4.97 | CA | VPC354* | 9 | 3.52 | CC |
| Reineta Pinta* | 12 | −0.04 | CC | **Amariega*** | 12 | 2.27 | CC |
| Campillo* | 12 | −0.65 | CC | VPC403* | 12 | 0.68 | CC |
| **Durón Encarnado*** | 10 | 4.58 | CC | **Corchu*** | 12 | 0.43 | CC |
| Lagar* | 10 | 0.28 | CC | | | | |

Bold font represents those accessions protected by the Protected Designation of Origin (PDO) “Sidra de Asturias”.
* Cultivars used in genome-wide association studies (GWAS).

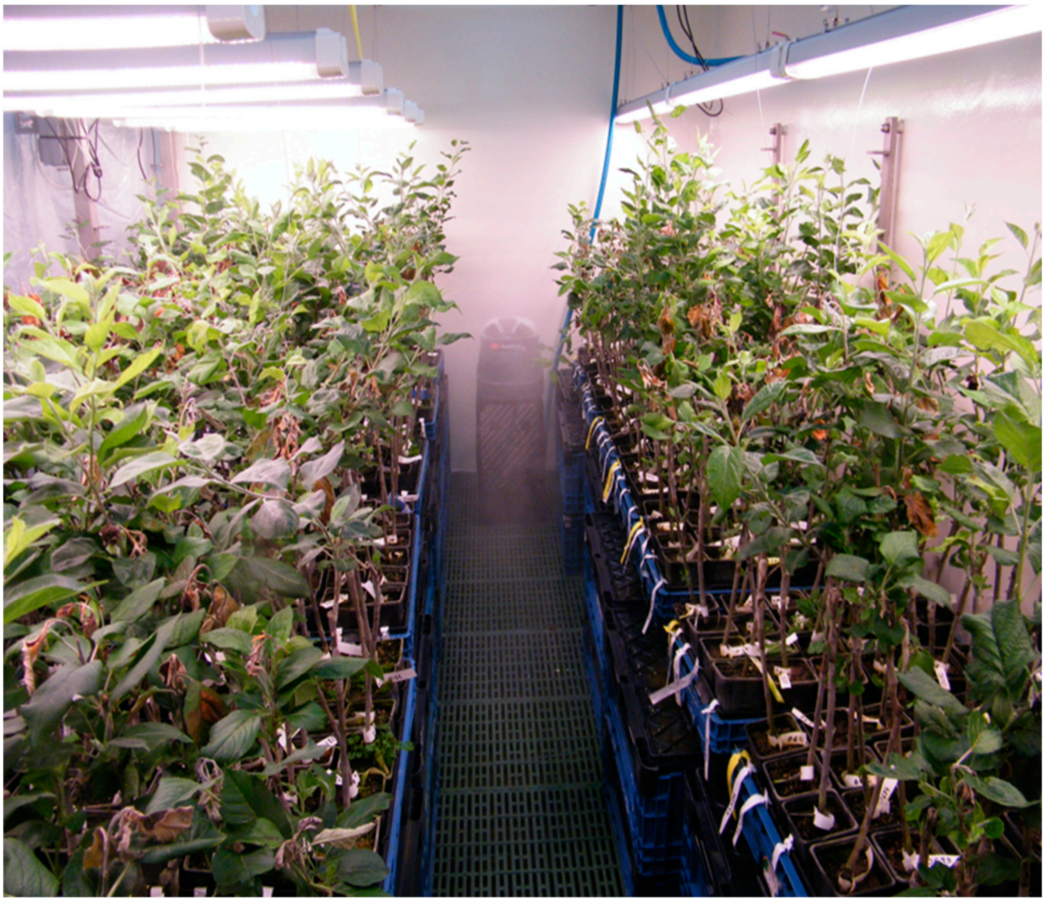

**Figure S1.** Image of potted plants 21 days after inoculation in the culture conditions of the high-biocontainment box (BSL3) at the IRTA - CReSA.
